# Complete Genome Sequences of Highly Arsenite-Resistant Bacteria *Brevibacterium* sp. Strain CS2 and Micrococcus luteus AS2

**DOI:** 10.1128/MRA.00531-19

**Published:** 2019-08-01

**Authors:** Shahid Sher, Abdul Rehman, Lars Hestbjerg Hansen, Tue Kjærgaard Nielsen

**Affiliations:** a Department of Microbiology and Molecular Genetics, University of the Punjab, Lahore, Pakistan; b Department of Environmental Science, Aarhus University, Roskilde, Denmark; c Department of Plant and Environmental Sciences, University of Copenhagen, Copenhagen, Denmark; University of Maryland School of Medicine

## Abstract

The complete genome sequences of two highly arsenite-resistant *Actinomycetales* isolates are presented. Both genomes are G+C rich and consist of a single chromosome containing homologs of known arsenite resistance genes.

## ANNOUNCEMENT

Arsenic is a toxic metalloid and is ubiquitously found in the environment. The amount of arsenic is increasing in our environment through two processes. Naturally, weathering of rocks, erosion of land, volcanism, etc., result in a release of arsenite. Anthropogenic activities, like combustion of fuels, the use of arsenic-containing pesticides, wood preservation, etc., lead to an increased release of arsenic into the environment. It is commonly found in the two forms arsenite and arsenate, with arsenite being 100 times more toxic because it can react with thiol groups of proteins. Microorganisms have developed mechanisms (e.g., oxidation, reduction, and methylation) for arsenic detoxification. By studying arsenic-resistant microorganisms, we can learn how to detoxify arsenic in the environment.

Brevibacterium sp. strain CS2 and Micrococcus luteus strain AS2 were isolated from industrial wastewater collected from Ittehad Chemicals in Kala Shah Kaku, Punjab, Pakistan. These bacterial strains were isolated by plating wastewater on lysogeny broth (LB) agar plates supplemented with 10 mM arsenite. The MICs for arsenite and arsenate were 40 mM and 280 mM for strain CS2 and 50 mM and 275 mM for strain AS2, respectively (Table 1). The optimum growth temperature and pH for both strains were 37°C and 7, respectively. Resistance against metals like Pb, Cd, Cr, Hg, Se, Co, and Ni was observed for both strains.

**TABLE 1:** MIC values of *Brevibacterium* sp. CS2 and Micrococcus luteus AS2 of tested metal ions

Metal ion (abbreviation)	MIC (mM) for strain:	
	*Brevibacterium* sp. CS2	Micrococcus luteus AS2
Arsenite (As^+3^)	40	50
Arsenate (As^+5^)	280	275
Lead (Pb)	4	5
Cadmium (Cd)	3	3
Chromium (Cr)	3	4
Mercury (Hg)	1	1.5
Selenium (Se)	5	5
Cobalt (Co)	5	5
Nickel (Ni)	4	3

AS2 and CS2 were inoculated in LB broth and incubated at 37°C for extraction of DNA using the MasterPure complete DNA and RNA purification kit (Lucigen, WI, USA). Illumina sequencing libraries were prepared using the Nextera XT sample preparation kit (Illumina, CA, USA) and were sequenced on an Illumina NextSeq 550 platform with a high-output kit v2.5 yielding 2 × 151-bp paired-end sequences. All programs were run with default parameters. Sequencing adapters and barcodes were trimmed with Cutadapt (v1.18) (1), and overlapping paired-end reads were merged with AdapterRemoval (v2.1.7) (2). A total of 1,174,861 and 1,298,092 read pairs were produced for CS2 (109× coverage) and AS2 (138× coverage), respectively. Sequencing libraries for Nanopore sequencing were prepared using the Rapid Barcoding Sequencing kit SQK-RBK004 and sequenced on a FLO-MIN106 flow cell on a MinION Mk1B device. Fast5 files were base called with Guppy (v2.1.3) (Oxford Nanopore), and Porechop (v0.2.4; https://github.com/rrwick/Porechop) was used for trimming of adapters and barcodes. Nanopore coverages were 387× and 325× for CS2 and AS2, respectively.

Long-read-only assemblies were performed with Flye (v2.4-release) (3) and subsequently polished with Illumina reads using unicycler_polish from Unicycler (v0.4.8-beta) (4). Each genome consists of a chromosome of 3.25 Mbp and 2.86 Mbp and G+C content of 70.1% and 72.8% for CS2 and AS2, respectively. Gene annotation with Prokka (v1.13.3) (5) predicted 2,682 and 2,950 coding sequences for AS2 and CS2, respectively. The taxonomy of the strains was determined by aligning the 16S rRNA genes to those of similar strains, and AS2 and CS2 show 99.0% and 97.3% similarity, respectively, to the 16S rRNA genes of Micrococcus luteus strain NCTC 2665 (NCBI RefSeq accession number NR_075062) and Brevibacterium senegalense strain JC43 (NCBI RefSeq accession number NR_118221). Both strains have genes related to arsenical resistance, including arsenate-mycothiol transferases and ACR3 family arsenite efflux pumps.

### Data availability.

The complete genome sequences of *Brevibacterium* sp. CS2 and Micrococcus luteus AS2 have been deposited in GenBank under accession numbers CP040020 and CP040019. Nanopore and Illumina raw read data have been uploaded to the SRA under BioProject numbers PRJNA540762 and PRJNA540761 for strains CS2 and AS2, respectively.

## References

[1] Martin M 2014 Cutadapt removes adapter sequences from high-throughput sequencing reads. EMBnet J 17:10–12. doi:10.14806/ej.17.1.200.

[2] Schubert M, Lindgreen S, Orlando L 2016 AdapterRemoval v2: rapid adapter trimming, identification, and read merging. BMC Res Notes 9:88. doi:10.1186/s13104-016-1900-2. 26868221 PMC4751634

[3] Lin Y, Yuan J, Kolmogorov M, Shen MW, Chaisson M, Pevzner PA 2018 Assembly of long error-prone reads using repeat graphs. bioRxiv. doi:10.1101/247148. 30936562

[4] Wick RR, Judd LM, Gorrie CL, Holt KE 2017 Unicycler: resolving bacterial genome assemblies from short and long sequencing reads. PLoS Comput Biol 13:e1005595. doi:10.1371/journal.pcbi.1005595. 28594827 PMC5481147

[5] Seemann T 2014 Prokka: rapid prokaryotic genome annotation. Bioinformatics 30:2068. doi:10.1093/bioinformatics/btu153. 24642063

